# Wrist redundancy management during pointing tasks remains stable over time and in presence of a visuomotor perturbation

**DOI:** 10.1038/s41598-023-33531-2

**Published:** 2023-04-26

**Authors:** Luigi Raiano, Alessia Noccaro, Giovanni Di Pino, Domenico Formica

**Affiliations:** 1 Unit of Neurophysiology and Neuroengineering of HumanTechnology Interaction (NeXT), Università Campus Bio-Medico di Roma, Via Álvaro del Portillo 21, 00128 Rome, Italy; 2 Neurorobotics Lab, School of Engineering, Newcastle University, Newcastle Upon Tyne, NE1 7RU UK

**Keywords:** Biomedical engineering, Dynamical systems

## Abstract

Pointing at a screen using wrist and forearm movements is a kinematically redundant task, and the Central Nervous System seems to manage this redundancy by using a simplifying strategy, named Donders’ Law for the wrist. In this work we investigated (1) whether this simplifying approach is stable over time and (2) whether a visuomotor perturbation provided in the task space influences the strategy used to solve the redundancy problem. We conducted two experiments asking participants to perform the same pointing task in four different days (first experiment), and providing a visual perturbation, i.e. a visuomotor rotation to the controlled cursor (second experiment), while recording their wrist and forearm rotations. Results showed that the participant-specific wrist redundancy management (described by the Donders’ surfaces) (1) neither changes over time (2) nor varies when a visuomotor perturbation is provided in the task space.

## Introduction

During everyday tasks, the Central Nervous System (*CNS*) faces motor redundancy while controlling voluntary movements^1–4^. A common example is controlling wrist and forearm rotations during pointing tasks. In fact, while this task requires to control only two degrees of freedom (DoFs), the *CNS* controls three DoFs, namely wrist flexion/extension (*FE*), radial/ulnar deviation (*RUD*) and forearm pronation/supination (*PS*)^5^. Previous studies have shown that the brain seems to manage the redundancy during pointing with the wrist by implementing a neural constraint, named *Donders’ Law for the wrist* for similarity to the empirical law originally described for eye movements by Donders in 1847^6^. In other words, the *Donders’ Law* poses that the three wrist and forearm rotations are constrained to lie on a two-dimensional surface, named *Donders’ surface*, which has been found to be * participant* specific and *volatile*^5,7–10^. The adjective *volatile* refers to the contingency that *Donders’ Law* can be violated and therefore considered as a “soft” constraint imposed by the brain, rather than a physical constraint due to a bio-mechanical characteristic of the wrist. Indeed, violations to *Donders’ Law* have been reported when participants were asked to perform pointing tasks attached to a wrist robot, which caused the Donders’ Surfaces to disappear^7^. Moreover, violations to *Donders’ Law* have been reported also for unconstrained movement of the human arm^11–13^ or for the eye when participants were instructed to move as fast as possible^14^.

However, some aspects of the *Donders’ Law* for the wrist still need to be further investigated. In fact, little is known about its stability over time (i.e. whether *Donders’ Surfaces* change over time within participants), and in response to external perturbations, such as visuomotor rotations in the task space during pointing tasks. As regards the stability of the redundancy management, it has been demonstrated that for arm movements aimed at pointing or grasping targets within a typical arm workspace, the final configuration of the arm does not depend on the starting position^15^. On the other hand, a preliminary study showed that visuomotor perturbation, provided to the controlled cursor, seems not to affect the specific implementation of the Donders’ Law^9^. Nevertheless, this work^9^ has some limitation in terms of protocol design, number of participants enrolled, and analysis, and no further studies about this topic have been carried out after.

In this work, we specifically study the stability of the *Donders’ Law* by using an interactive clock-like video-game where participants are asked to point at targets on a screen using only the wrist and forearm rotations.

In particular, two different experiments (described respectively in “Assessing Donders’ law stability over time“ and “Assessing Donders’ law during motor adaptation“) have been conducted on two groups of participants: (1) the first one focused on the stability over time of the (Donders’ Law) for the wrist, testing whether it changes within participants over four different days; (2) the second experiment aimed at studying the *Donders’ Law* for the wrist in presence of a visuomotor perturbation during the pointing tasks. To this aim, we analyzed in each experiment the following parameters: (1) the task-space accuracy in performing the pointing movements in terms of deviation from an ideal straight path connecting the staring position and the target one; (2) the reliability of the estimated *Donders’ Surfaces* in terms of their *thickness*^5^ and (3) the participant-specific implementation of the *Donders’ Law* during the tasks in terms of *Shape Index* of the estimated *Donders’ Surfaces*^8^.

## Results

### Assessing Donders’ law stability over time

**Figure 1:**
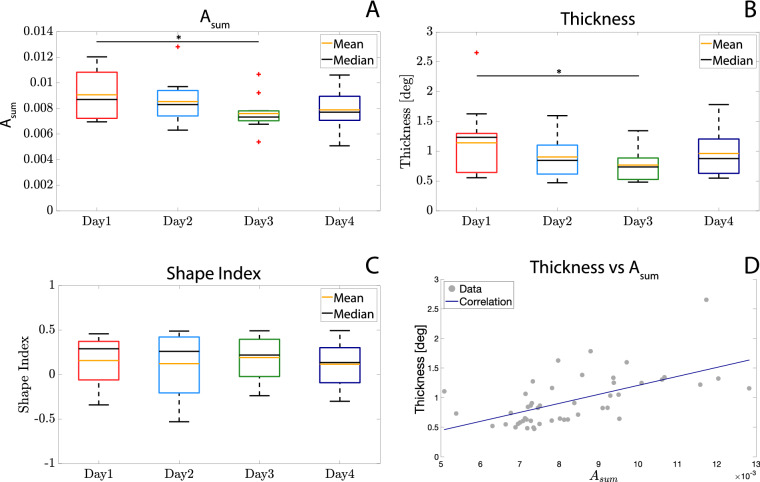
(**A**) Average Task-Space Accuracy $A_{sum}$ (along trials and participants) over the 4 days; (**B**) average *Thickness* (along trials and participants) over the 4 days, neglecting the discarded outliers; (**C**) *Shape Index* averaged along trials and participants over the 4 days, neglecting outliers; (**D**) correlation between *Thickness* and *Task-Space Accuracy*. Pearson’s $R = 0.62$ with a $p = 3.8 \times 10^{-6}$. Within box-plots (**A**–**C**), red crosses represent the data labelled as outliers by the MATLAB™ function *boxplot*, net of neglected outliers on the basis of *Thickness* analysis.

Figure 1A,B shows the $A_{sum}$ and thickness values averaged along trials and participants in the four different days. All Thickness values are below 3$^{\circ }$, congruently with previous studies^8^. Although both indices significantly vary over days ($A_{sum}$: p = 0.04, F = 4.49; *thickness*: p = 0.03, F = 3.24), post-hoc analysis reveals significant differences only between *Day 1* and *Day 3* ($A_{sum}$: p = 0.01, t = 3.4; *Thickness*: p = 0.03, t = 3.07). We also found a significant correlation (Spearman’s correlation $\rho$ = 0.62, p = 3.82 $\times 10^{-6}$, see Fig. 1D) between such parameters, as suggested by their similar behaviors over sessions. We implemented the correlation considering all trials (except for the outliers).

Shape index (tested with a 1-way ANOVA considering participants as independent factor) showed significantly different values along participants (p = 1.15 $\times 10^{-54}$, K = 281.68), confirming that the peculiar shape of the Donders’ surface is participant-specific, according to previous studies on this topic^7–9^. On the other hand, as depicted in Fig. 1C), the RM-ANOVA revealed that *Shape Index* did not change within participants over days (p = 0.74, F = 0.28, Greenhouse–Geisser sphericity correction method was used).

### Assessing Donders’ law during motor adaptation

**Figure 2:**
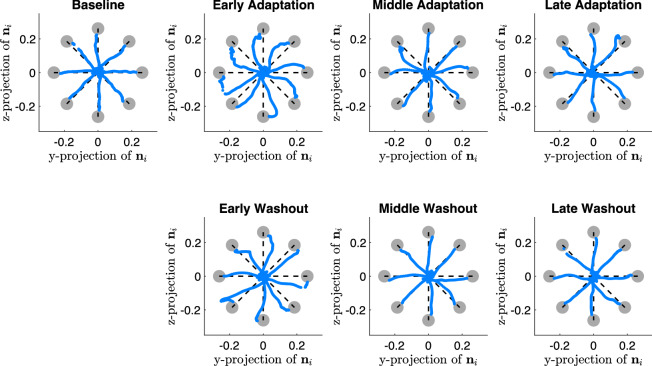
Pointing trajectories for a representative participant, showing *baseline*, *adaptation* (divided into early trials 1 $\div$ 10, middle trials 11 $\div$ 25 and late trials 26 $\div$ 40) and washout (divided into early, middle and late trials). Blue dots represent movements from center to peripheral targets, while grey circles represent the targets that the participants are asked to point. Noteworthy, the trajectories depicted for the adaptation phases are rotated according to the provided visuomotor perturbation, i.e. 30$^{\circ }$ CCW rotation, in order to represent the participant perspective during the task execution.

**Figure 3:**
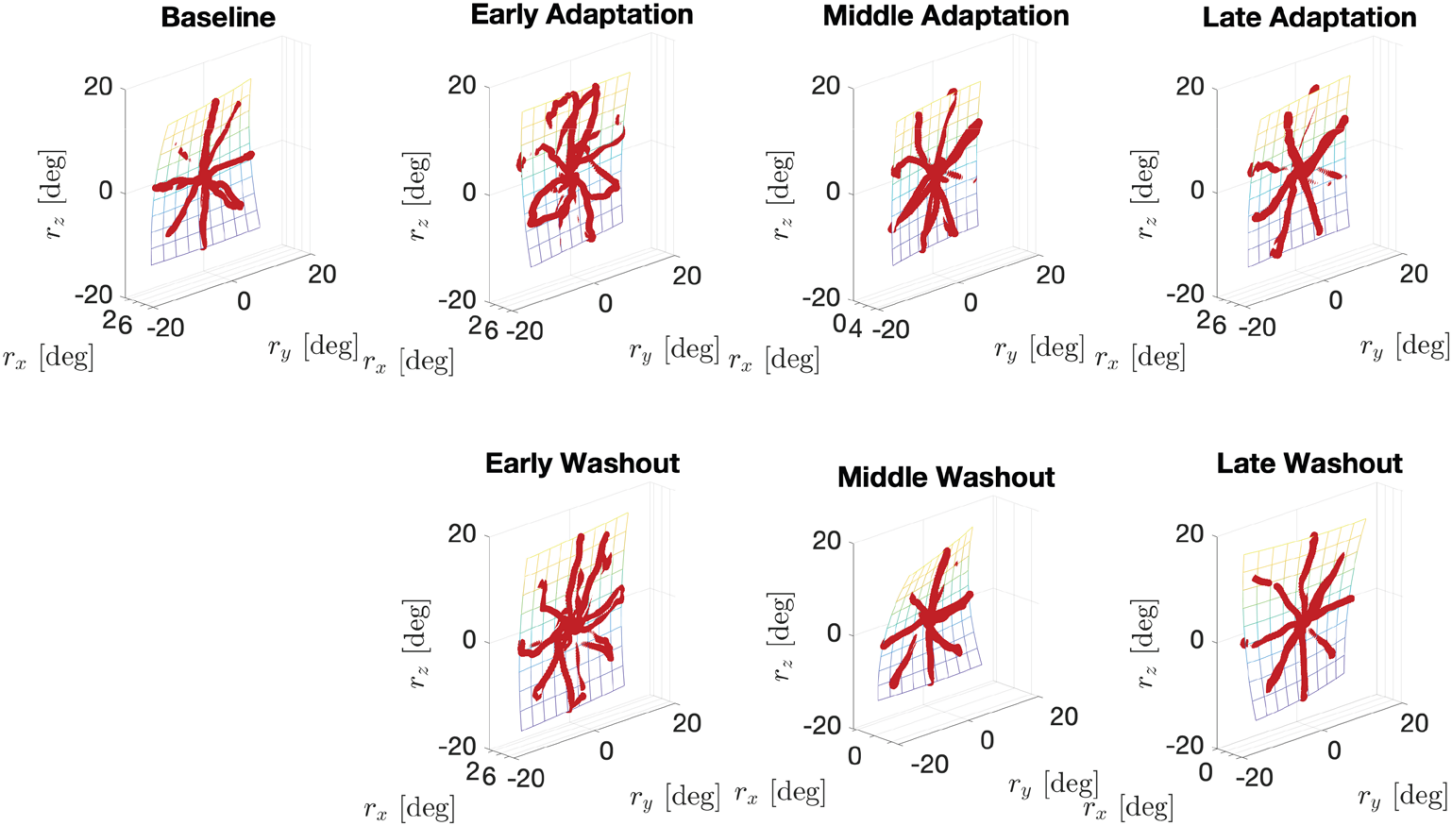
Donders’ surfaces for a representative participant, showing *baseline*, *adaptation* (divided into early trials 1 $\div$ 10, middle trials 11 $\div$ 25—and late trials 26 $\div$ 40) and washout (divided into early, middle and late trials). Red dots represent recorded wrist rotations.

Figure 2 shows the pointing trajectories over sessions for a representative participant, while the associated Donders’ surfaces are reported in Fig. 3.

All the parameters described in “Data analysis“ were found to be normally distributed, thus we used RM-ANOVA and t-test (for implementing post-hoc analysis) in all cases except for $\tau _{sum}$ (adaptation time), for which we employed the Wilcoxon Signed-Rank test. Corrections in post-hoc tests were implemented using the Bonferroni correction.

**Figure 4:**
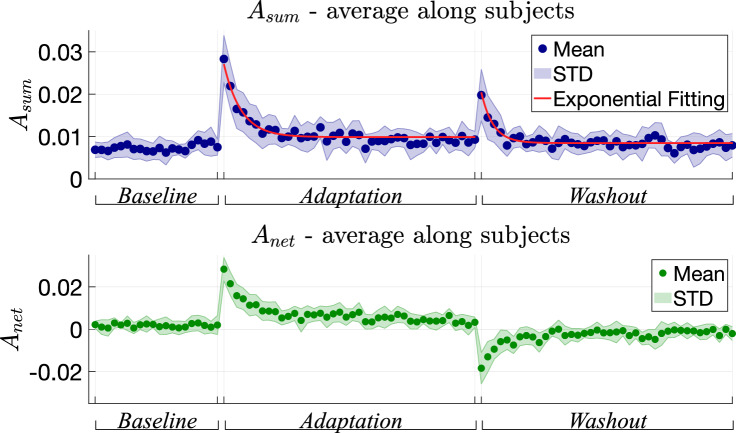
Behaviour of $A_{sum}$ and $A_{net}$ over trial. Moreover, the exponential fitting curve is superimposed on $A_{sum}$ data.

**Figure 5:**
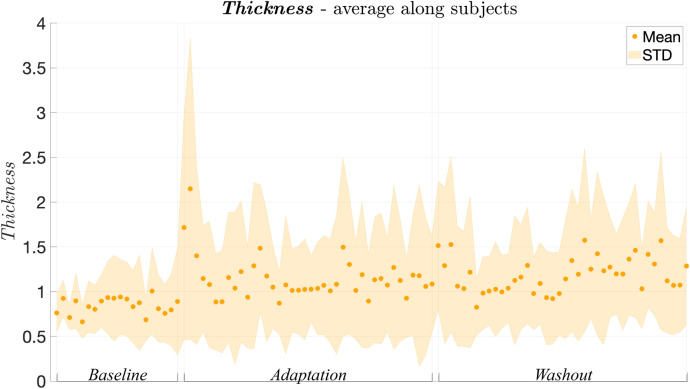
Thickness averaged along participants over trials.

**Figure 6:**
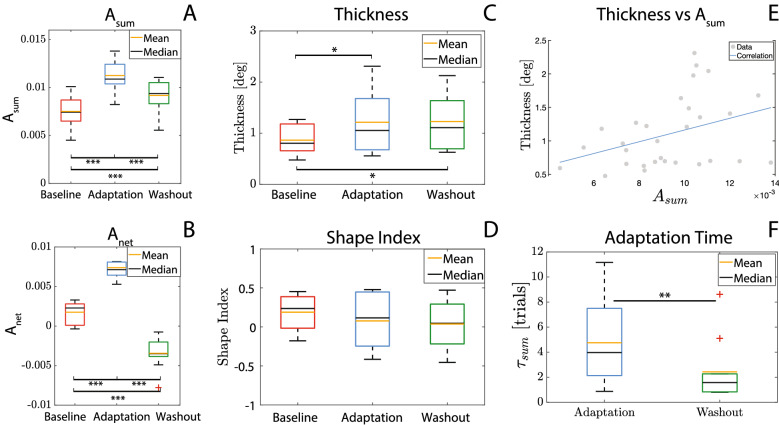
(**A**–**D**) Performance parameters averaged along trials and participants in baseline, adaptation and washout phases. (**A**) $A_{sum}$; (**B**) $A_{net}$; (**C**) thickness; (**D**) Shape Index; (**E**) correlation between thickness and $A_{sum}$. (**F**) Adaptation time $\tau _{sum}$ averaged along trials and participants in adaptation and washout phases.

The results related to $A_{sum}$ and $A_{net}$ averaged for all participants and over trials are reported in Fig. 4. Both in adaptation and washout phases, $A_{sum}$ and $A_{net}$ exhibits a sudden variation in the first trials with respect to the end of the previous one, where they both settle with training. While $A_{sum}$, which is the absolute error and therefore always positive, increases at the beginning of both *adaptation* and *washout* phases, $A_{net}$ shows a variation with opposite signs at the beginning of the *adaptation* and *washout* phases, indicating that participants deviate in opposite directions when the visuomotor rotation is introduced and then removed.

As visible from Fig. 6A,B, both $A_{sum}$ and $A_{net}$ statistically vary over sessions ($A_{sum}$: p = 1.89 $\times 10^{-7}$, F = 41.27; $A_{net}$: p = 9.17 $\times 10^{-13}$, F = 186.78, Greenhouse–Geisser sphericity correction was implemented). Post-hoc tests reveal that the absolute deviation from the straight line ($A_{sum}$) is significantly higher during *Adaptation* than during *Washout* (p = 1.70 $\times 10^{-4}$, t = 5.04). In both phases it is higher than *Baseline* values (*baseline* *vs* *adaptation* p = 1.18 $\times 10^{-7}$, t = − 9.06; *baseline* *vs* *washout* p = 7.93 $\times 10^{-4}$, t = − 4.02). Similar results were found for $A_{net}$ (*baseline* *vs* *adaptation* p = 2.49 $\times 10^{-8}$, t = − 10.05; *baseline* *vs* *washout* p = 8.42 $\times 10^{-8}$, t = 9.27; *adaptation* *vs* *washout* p = 5.23 $\times 10^{-13}$, t = 19.32), where the *washout* phase shows negative values, highlighting the presence of *aftereffects* of the adaptation to visuomotor rotation, i.e. a deviation in the opposite direction with respect to the *adaptation* phase when the perturbation is removed.

As regards the *Thickness*, according to the behaviour over trials depicted in Fig. 5 and the boxplots of Fig. 6C, it ranges from 0.48$^{\circ }$ to 1.27$^{\circ }$ in *Baseline*, from 0.56$^{\circ }$ to 2.31$^{\circ }$ in *Adaptation* and from 0.63$^{\circ }$ to 2.13$^{\circ }$ in *Washout*, congruently with previous studies^5,9,10^ and the results obtained in the first experiment (see “Assessing Donders’ law stability over time"). Moreover, *Thickness* significantly differs over sessions within participants (p = 5.35 $\times 10^{-5}$, F = 7.09) and post-hoc tests revealed a significant increase with respect to the *Baseline* both in *Adaptation* (p = 0.01, t = − 3.20) and in *Washout* (p = 0.01, t = − 3.32) phases. Conversely, no statistically significant difference was found between *Adaptation* and *Washout*.

Similarly to the data analysis presented in “Assessing Donders’ law stability over time“, we found a significant correlation between $A_{sum}$ and *Thickness* (p = 0.03, $\rho$ = 0.4, see Fig. 6E). In this case values were averaged along trials of each session, and all data from all participants were grouped, to run the correlation by using the Spearman’s technique.

The number of trial outliers discarded in all participants for each session are reported in Table 2.

As regards the *Shape Index*, also in this experiment, it was found to be significantly different over the participants (p < 0.0001, Z = 565.12 by using a Kruskal–Wallis test), while no statistical difference has been found over the sessions (p = 0.12, F = 2.75, Greenhouse–Geisser sphericity correction method was applied).

Finally, the adaptation time $\tau _{sum}$, expressed as number of trials (see Fig. 6F), is significantly higher in the *adaptation* phase than in the *washout* (p = 5.86 $\times 10^{-3}$, W = 53), showing that the adaptation phase to a visuomotor perturbation takes longer than re-adapting to the baseline condition. In Fig. 4, the exponential fitting of the average $A_{sum}$ is also reported, superimposed to the recorded data.

## Discussion

### Stability over time

The decreasing trend of $A_{sum}$ over days may suggest a learning effect, despite the task involves the management of the wrist redundancy. A similar reduction over days of the *Thickness* suggested a possible correlation between the two parameters, confirmed by the significant Spearman’s correlation coefficient equal to 0.62. This indicates that the more participants learn the task, the better they implement the redundancy solution. Indeed, reducing the error in the task space and improving the management of the redundancy (in terms *thickness* of *Donders surfaces*) may compete in an iterative and concurrent way in the wrist coordination: the more the participants become confident with the task, the higher the accuracy in managing the redundancy. Such evidences are in line with the most popular theories of human motor control, in which the motor output is the result of a cooperative and iterative process for optimizing all factors involved, *i.e.* the accuracy in performing the task and the management of redundancy^16–18^.

Concerning the implemented strategy, i.e. the participant-specific shape of *Donders surface*, we verified that it depends on the single participant, thus denoting a personal motor style. In addition, we found it to be stable over time, since no statistical differences have been found over days. Such results confirmed that the *Donders’ Law*, expressed through the *Shape Index*, could be considered as a *personal motor sign* and a reliable parameterization to model the participant’s specific implementation of the redundancy problem^5^.

### Motor adaptation

The analysis carried out on the task space accuracy (*i.e.* $A_{sum}$ and $A_{net}$), revealed that despite the task involves the management of the kinematic redundancy, motor learning does occur. Indeed, (see Figs. 4,  6) when the visuomotor perturbation is provided (and when removed), during the first trials participants show a sudden decrease of their performance in terms of path error. However, training over several trials allows participants to learn a new internal model, leading to an improvement of their performance. Moreover, results show that participants take longer to learn a new internal model, i.e. pointing with a visuomotor perturbation, than re-adapting to the baseline condition. Indeed, the estimated adaptation time related to the adaptation session is significantly higher than the one related to the washout (see Fig. 6).

Nevertheless, when considering the wrist redundancy management, the participant-specific shape of *Donders’ surfaces* is preserved during both the adaptation and washout phases, even if the thickness is slightly increased with respect to baseline values. Anyway, it remains in all cases below 3$^{\circ }$, which is a threshold largely consistent with previous studies^9^ and with the results obtained from our first experiment (see “Assessing Donders’ law stability over time“).

Such results confirm that even though the task requires a management of the wrist redundancy, motor learning still occurs similarly to classical and widely studied problems of adapting to a constant perturbation^19–21^ and confirming the preliminary findings presented in^9^.

However, the result that, during both *Adaptation* and *Washout* phases, the *Thickness* is higher than the *Baseline* (see Fig. 6), suggests that the presence of the visuomotor perturbation influences how precisely participants implement the *Donders’ Law*. A possible explanation may underlie in the hierarchical processes that occur during motor learning^22–24^: learning how to adapt to a perturbation in the task space may have a higher priority than managing the redundancy. Specifically, when participants face with a task which they have to adapt, the brain might be more focused in reducing task space error rather than optimally managing the redundancy problem.

On the other side, the stability of the *Shape Index* over sessions suggests that the specific policy used by each participant to combine wrist DoFs for solving the redundancy does not change when participants have to adapt to a task-space visuomotor perturbation. Although the redundancy solution is less precise (given the increase of *thickness* values), the participant-specific policy used to control the wrist in a kinematically redundant task is not disrupted, confirming preliminary results from^9^. Thus, the *Shape Index*, being personal and stable over days and in presence of visuomotor perturbations, may be denoted as a suitable parameter for describing how the control policy is implemented in each participant during redundant pointing tasks with the wrist.

Although the redundancy management can be considered as a *soft constraint*, *i.e.* it is not due to any physical constraint since it can be violated in some conditions^7,10^, it could be strictly correlated to the participant specific muscle-skeletal conformation and thus to participant specific bio-mechanics^9,25^. In other words, due to participant specific bio-mechanics (e.g. wrist impedance), the observed behaviour might be attributed to a side effect of a control strategy applied to FE and RUD^25^. Therefore, *Shape Index* can be ultimately defined as a participant-specific motor sign and eventually used as bio-marker for neurological diseases that involve neuro-muscular or muscle-skeletal disorders, such as Parkinson’s Disease^26^ or job-related disorders^27^. Future works will target to specific studies on participants with these diseases in order to further investigate this aspect.

## Conclusions

In this work we studied the stability of the wrist redundancy management during pointing tasks focusing on two different aspects: (1) whether the *Donders’ Law* is stable over days for a single individual (see “Assessing Donders’ law stability over time“) and (2) whether it is influenced by a visuomotor perturbation provided in the task space (see “Assessing Donders’ law stability over time“). We enrolled two groups of participants and we studied the two scientific questions separately. We found a significant correlation between the accuracy in the task space ($A_{sum}$) and and the accuracy in implementing the Donders’ Law (*Thickness*), suggesting an iterative and concurrent influence of the task performance and redundancy management in the wrist coordination.

Mainly, we found that the *Shape Index*, that defines the peculiar shape of the Donders’ Surface (i.e. the geometrical representation of the Donders’ Law) is significantly consistent for each participant over days. This finding suggests that Donders’ Law can be considered as a reliable model to describe redundancy control policy.

On the other hand, we also demonstrated that redundancy solution policy persists even when participants have to adapt to a visuomotor perturbation and the participant-specific control policy implemented, characterized by the *Shape Index*, does not change across sessions during both adaptation and washout phases.

Such results suggest that *Shape Index* may be also used as bio-marker for neuromuscular or muscle-skeletal disorders. To this aim, future works should investigate wrist redundancy management during pointing tasks in participants affected neuro-muscular diseased, such as Parkinson’s Disease, in order to define novel protocols for assessing the motor symptoms of the pathology and optimizing its treatment^28,29^. In addition, we will also test pointing tasks with the wrist during non-invasive neuromodulation procedures, e.g. transcranial Direct Current Stimulation (tDCS)^30^ or robot-aided Transcranial Magnetic Stimulation (TMS)^31^, in order to investigate which are the brain areas mainly involved in managing the redundancy solution of the wrist.

## Materials and methods

### Experimental setup

**Figure 7:**
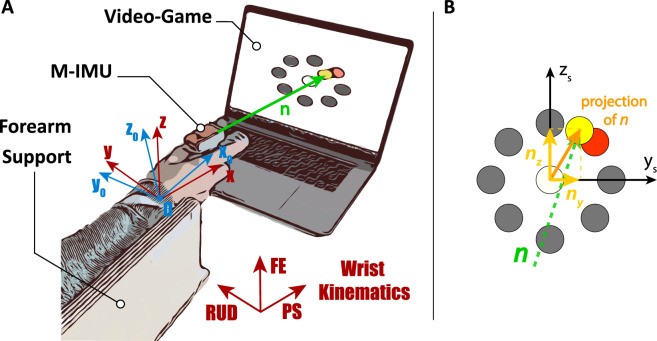
(**A**) Overview of the experimental setup. For details on wrist kinematics refer to^8^. The blue reference frame “0”, centered in the wrist center of rotation, refers to the neutral position of the wrist-*zero position*- and corresponds to the initial position; the red reference frame represents the current wrist orientation recorded by the M-IMU. (**B**) Scheme of the clock-like game with the 2D projection of the pointing vector *n* onto the screen reference frame $\{y_s, \, z_s\}$.

Participants were seated in front of a monitor, with the arm fixed on a support and straps used to minimize torso, shoulder and elbow movements, so that only wrist rotations and forearm pronation-supination were left unconstrained. Wrist rotations were measured with a Magneto-Inertial Measurement Unit (M-IMU, MTw Awinda by XSens Inc.) mounted on top of an ergonomic hand-held device, which provides rotation matrices of the handle with respect to an external reference frame. The M-IMU, wirelessly connected to a laptop, was configured to continuously record the sequence of rotation matrices ($\varvec{R}_i$ related to the *i*-th samples) with a sampling rate equal to 100 Hz. Before starting, the wrist was oriented in its neutral configuration, hereinafter denoted as *zero position*^32^, which was used as fixed reference frame for evaluating relative rotations while performing the pointing task^5,9^. The complete setup is represented in Fig. 7A.

### Pointing task

Pointing tasks were implemented through an interactive MATLAB^TM^-based video-game, in which participants could see the pointed target in real-time and control the cursor position with wrist rotations. For a generic orientation of the hand expressed by the rotation matrix $\varvec{R}_i$, the *pointing vector* ($\varvec{n}$) can be computed as follows:

1 $$\begin{aligned} \varvec{n}_i = \varvec{R}_i [1,0,0]^T \end{aligned}$$

During the task, the *i*-th pointing position (second and third components of $\varvec{n}_i$) projected onto the monitor plane was evaluated in real-time and provided to the user as visual feedback (see Fig. 7B).

Participants were instructed to move the cursor on the screen towards eight randomly highlighted peripheral targets. The position of the targets was designed such that participants are required to rotate the wrist of 15$^{\circ }$ to hit them, corresponding to a linear distance travelled by the cursor of about 10 cm. Distance between the monitor and the center of rotation of the wrist (point 0, see Fig. 7) was the same for all participants. Participants have a 2 s timeout to reach a peripheral target, and as soon as it is reached, the central target is highlighted so that Participants can move back into the initial configuration (without any timeout). Once the central target is then reached, the next peripheral target is highlighted and the trial goes on until its completion. In addition, a target is considered *reached* if the distance between the current position of the cursor and the target itself is smaller than 0.03 rad. To inform the participants, the reached target is unlighted. A single trial is completed once the participants reached all eight peripheral targets.

## Experimental protocols

We conducted two experiments on two different groups of participants, to separately assess the *Donders’ Law* in terms of stability over time and in presence of visuomotor perturbation. The two experimental protocols are described in the following sections, together with the statistical analysis used for the two datasets. We used the software JASP^33^ to perform all statistical tests and we selected the significance threshold equal to 0.05 in all cases. Both studies were approved by the Ethics Committee of Universita’ Campus Bio-Medico di Roma (EMBODY protocol) and carried out in accordance with the Declaration of Helsinki and following amendments. All participants gave written informed consent before the experiments. Right handedness for all participants was verified by means of an Oldfield test^34^ (considering a score higher than +5), which has been administered to the participants before starting the experiments.

### Assessing Donders’ law stability over time

Participants were asked to complete a series of pointing tasks with their right wrist in four different days, at least 1 week apart. In each session day, participants performed 30 trials: first 10 trials were used to let the participants familiarize with the system and thus were not included in the statistical analysis.

#### Participants

Twelve right-handed healthy volunteers (4 male and 8 female, aged between 21 and 34 years old), with neither history of neuromuscular disorders nor previous wrist injuries, participated to the experiment.

### Assessing Donders’ law during motor adaptation

Participants were asked to perform the pointing task in a single session, divided into four phases:

1. *Familiarization*: 5 trials used to let the participants become confident with the proposed setup and not considered in data analysis;
2. *Baseline*: 20 trials, considered as basis values for each participant;
3. *Adaptation*: 40 trials, in which a visuomotor perturbation is presented as a 30$^{\circ }$ counter clockwise (CCW) visual rotation of the controlled cursor (*i.e.* the position pointed by the participants is displayed as rotated of 30$^{\circ }$ with respect to the real one);
4. *Washout*: 40 trials, in which the visuomotor perturbation is removed.

#### Participants

Ten right-handed volunteers (4 male and 6 female, aged between 19 and 30 years old) with neither history of neuromuscular disorders nor previous wrist injuries, completed the experiment. It is worth noting that the group of enrolled participants for this experiment is different from the one tested in “Assessing Donders’ law stability over time“.

### Data analysis

Given the *i*-th hand orientation ($\varvec{R}_i$) retrieved by the M-IMU, it is possible to determine the wrist axis and amount of rotation by using the rotation vector ($\varvec{r}_i$) as follows^5,7–9^:

2 $$\begin{aligned} \varvec{r}_i = \begin{bmatrix} r_{ix} \\ r_{iy} \\ r_{iz} \end{bmatrix}= \frac{1}{1 + R_{i1,1} + R_{i2,2} + R_{i3,3} } \begin{bmatrix} R_{i3,2} - R_{i2,3} \\ R_{i1,3} - R_{i3,1} \\ R_{i2,1} - R_{i1,2} \end{bmatrix} \end{aligned}$$

Despite pointing at virtual targets is a 2-dimensional task, the rotation vector belongs to 3-dimensional space. Previous studies demonstrated that $r_{ix}$, $r_{iy}$ and $r_{iz}$ lie on a 2-dimensional surface embedded in the 3D space of general rotations, named *Donders’ Surface*^5,9,10^, which can be estimated by means of a least-squares approximation (implemented using the MATLAB function “*nlinfit*”) according to the following model:

3 $$\begin{aligned} {\tilde{r}}_{ix} = C_1 + C_2r_{iy} + C_3r_{iz} + C_4r_{iy}^2 + 2C_5r_{iy}r_{iz} + C_6r_{iz}^2, \end{aligned}$$

where $C_1$–$C_6$ denote the coefficients computed by fitting procedure. Further details can be found in^7^.

In order to assess the task performance and the stability of the Donders’ Law both over time and in presence of visuomotor perturbation, we calculated the following parameters:

1. $A_{sum}$–$A_{net}$: path deviation from a straight line connecting the starting position to a peripheral target for each movement^9,35^, declined as absolute error ($A_{sum}$) and relative deviation to the left or the right of the straight path ($A_{net}$)^35^. Such indices quantify the task performance in terms of error with respect to the straight path. Further details about $A_{sum}$ and $A_{net}$ derivation can be found in^35^.
2. *Thickness*: the standard deviation of the error between the fitted surface and measured first component of the rotation vector ($r_{ix}$). *Thickness* values denote how well the participants implement the Donders’ Law: the lower the thickness, the better the Donders’ surface predicts the recorded data.
3. *Shape Index*: being the *Donders’ Law* geometrically represented by a 3D surface, the *Shape Index* characterizes the specific shape of the Donders’ surface, thus representing the personal motor style of each participant in implementing the redundancy solution. It is defined as follows^8^:
  4 $$\begin{aligned} Shape \ Index = \frac{2}{\pi }atan2(H,\sqrt{H^2 - K}) \end{aligned}$$
  denoting *H* and *K* the *Mean Curvature* and the *Gaussian Curvature* of the Donders’ surface respectively^8,36^. Further details can be found in^8^.
4. *Adaptation Time* ($\tau _{sum}$): the time required for the participant to adapt to the visuomotor rotation (or to its removal during the washout phase), computed by fitting $A_{sum}$ over trials with an exponential curve as $A_{sum}[t] = A_{sum}^{0} e^{\frac{-t}{\tau _{sum}}} + A_{sum}^{\infty }$, where $A_{sum}^{0}$ and $A_{sum}^{\infty }$ denote the value at time zero and at steady state, assuming a single-state learning model^37,38^. This parameter is only used when the participants are adapting or de-adapting to the visuomotor rotation provided in the second experiment.

All parameters related to the task performance were computed considering only the movements from center to peripheral targets, since movements toward the wrist neutral position result to be easier for participants than moving toward periphery^35^. Conversely, for the computation of the Donders Surfaces, and thus all related parameters, the entire movement was used (i.e. from center to periphery and vice versa). *Thickness* was used to identify and exclude outlier trials from statistical analysis (trials with *Thickness* higher than the average over all trials plus 2 standard deviations have been excluded). All the above-mentioned parameters have been evaluated in each participant for each trial, and then averaged along the total amount of trials, after excluding outliers. Specifically, the results related to outlier exclusion of the first experiment (“Assessing Donders’ law stability over time“) are reported in Table 1, while the ones related to the second experiment (“Assessing Donders’ law during motor adaptation“) are reported in Table 2.

**Table 1:** Number of trial outliers discarded in all participants for each day of the first experiment on the basis of the computed *Thickness*.

	Day 1	Day 2	Day 3	Day 4
N. of trial outliers	17	16	15	16
Percentage of trial outliers	7.08%	6.67%	6.25%	6.67%

The *Thickness* threshold was set to the average value plus two times the standard deviation.

**Table 2:** Number of trial outliers discarded in all participants for each session of the second experiment on the basis of the computed *Thickness*.

	Baseline	Adaptation	Washout
N. of trial outliers	14	26	22
Percentage trial outliers	7%	6.5%	5.5%

The *Thickness* threshold was set to the average value plus two times the standard deviation.

### Statistical analysis

We firstly checked for the normality of the data by means of the Shapiro-Wilk test^39^. As regards the assessment over time, according to the results of the normality test, we run either 1-Way Repeated Measures Analysis Of Variance (RM-ANOVA) or 1-Way Friedman Test considering the factor “Day” as the 4-levels within participants independent variable.

Concerning the assessment during motor adaptation, we run either a parametric (Repeated Measure ANOVA) or non-parametric (Friedman Test) 1-Way Repeated Measures Analysis, separately on each parameter ($A_{sum}$, $A_{net}$, *Thickness* and *Shape Index*), with the phases as 3-level factor (*i.e.* *baseline*, *adaptation* and *washout*). Moreover, we checked for the difference between *adaptation* and *washout* phases for $\tau _{sum}$ using a paired sample test.

## Data Availability

The datasets generated and/or analysed during the current study are not publicly available since the informed consent signed by the volunteers enrolled in the study did not contain the possibility to share the data publicly. Nevertheless, data are available from the corresponding author on reasonable request.
